# Evaluation of Cardiometabolic Parameters among Obese Women Using Oral Contraceptives

**DOI:** 10.3389/fendo.2017.00256

**Published:** 2017-09-29

**Authors:** Júnia Raquel Dutra Ferreira, Milena Magalhães Aleluia, Camylla Vilas Boas Figueiredo, Larissa Castro de Lima Vieira, Rayra Pereira Santiago, Caroline Conceição da Guarda, Cynara Gomes Barbosa, Ricardo Riccio Oliveira, Elisângela Vitória Adorno, Marilda de Souza Gonçalves

**Affiliations:** ^1^Instituto Gonçalo Moniz/FIOCRUZ, Salvador, Brazil; ^2^Faculdade de Farmácia, Departamento de Análises Clínicas e Toxicológicas, Universidade Federal da Bahia, Salvador, Brazil

**Keywords:** combined oral contraceptives, obesity, cardiometabolic parameters, women, C-reactive protein, high-density lipoprotein cholesterol, cardiovascular risk

## Abstract

**Background:**

Combined oral contraceptive (COC) use has been associated with an unfavorable impact on carbohydrate and lipid metabolism in diverse populations of normal weight and obese women. The present study aimed to evaluate the cardiometabolic and inflammatory profiles of women in northeastern Brazil with respect to COC use and obesity.

**Methods:**

We performed a cross-sectional study to verify cardiovascular parameters, including blood pressure (BP), fasting serum glucose, lipid, and inflammatory profile, in a population of women aged 15–45 years, considering obesity and COC use. Our sample consisted of 591 women, 481 women who were COC users, and 110 age-matched women who were COC non-users, classified as obese and non-obese according to BMI.

**Results:**

COC use and obesity were associated with increased systolic (*p* ≤ 0.001) and diastolic BP (*p* = 0.001), blood glucose (*p* ≤ 0.001), total cholesterol (*p* = 0.008), low-density lipoprotein cholesterol (*p* ≤ 0.001), very low-density lipoprotein cholesterol (*p* ≤ 0.001), triglycerides (*p* ≤ 0.001), ferritin (*p* = 0.006), C-reactive protein (CRP) (*p* ≤ 0.001), and nitric oxide metabolites (*p* ≤ 0.001), as well as decreased high-density lipoprotein cholesterol (HDL-c) (*p* ≤ 0.001) in comparison to controls. CRP and HDL-c levels in obese COC users were determined to be outside reference range values. The odds of having lower levels of HDL-c and elevated CRP increased among obese COC users. COC use was independently associated with low levels of HDL-c, especially second-generation progestins (*p* < 0.001; OR = 8.976; 95% CI 2.786–28.914).

**Conclusion:**

Obesity and COC use were associated with alterations in lipid and inflammatory cardiometabolic parameters, particularly increased CRP levels and decreased HDL-c, which are considered markers of cardiovascular disease (CVD) risk. Given the need to prevent unintended pregnancy among obese women, together with weight loss counseling, it is important to evaluate the most effective and safest contraceptive methods to avoid the potential risk of developing CVD.

## Introduction

Since its introduction in the early 1960s, hormonal contraception has been widely used by millions of women to prevent pregnancy. Among the hormonal methods developed, combined oral contraceptives (COCs) are the most commonly used worldwide, representing the first choice contraceptive in the developed world and the third in developing countries (1–3).

Formulations of COCs containing high doses of both estrogen and progestin have changed over the years to reduce the levels of both compounds as a result of association with cardiovascular side effects and venous thromboembolism (VTE) (4). Nonetheless, the risk of arterial and venous thrombosis continues to remain associated with COC use.

Several genetic and environmental cardiovascular risk factors have also been associated with arterial and venous thrombosis, such as obesity, smoking and family medical history, among others. Increasing rates of obesity constitute a worldwide health concern due to the association with cardiovascular diseases (CVD), diabetes, high blood pressure (BP), altered cholesterol levels, and other disorders (5, 6). More than 50% of obese individuals around the world are concentrated in just 10 countries, including Brazil (7). The weight profile of the Brazilian population has changed in the recent decades, with a resulting increase in obesity. Among adult Brazilian women, the estimated prevalence of obesity is 20.6%. Obesity not only affects the overall health of the Brazilian population but also increases the need for annual investment in health care, especially the treatment of heart disease, stroke, and diabetes (8, 9). Obesity has been reported as a risk factor for both arterial and venous thrombosis because of its many effects on inflammation, coagulation and fibrinolysis (5, 6, 10, 11). In addition, COC use has also been shown to increase the risk of VTE by approximately twofold, and the risk of arterial thrombosis (myocardial infarction and stroke) by approximately threefold (10, 11).

Since it is plausible to assume that the use of COCs by obese women may present unfavorable alterations in cardiometabolic parameters, we performed a cross-sectional study to analyze the biochemical and inflammatory parameters of a Brazilian population, especially obese women who were COC users.

## Materials and Methods

### Study Design

A cross-sectional study was performed in women residing in the state of Bahia, Brazil, who sought routine examinations from August 2013 to July 2014 at a clinical laboratory health service provided by the College of Pharmaceutical Sciences, Federal University of Bahia (UFBA). We included a total of 591 women aged 15–45 years: 481 had been taking COCs for at least 3 months (study group), and 110 were age-matched non-COC users for at least 2 years (control group). Women were classified as either obese (OB) (BMI ≥ 30 kg/m^2^) or non-obese (NOB) (BMI < 30 kg/m^2^). The exclusion criteria were: surgery or immobilization within 6 months prior to study inclusion; previous history of hysterectomy; menopause; clinical diagnosis of bacterial or viral infection; pregnancy, or delivery 4 months prior; lactation; use of any other hormonal contraceptives or hormonal therapy; the presence of malignant or autoimmune disease. In addition, we excluded women who were using COCs for medical reasons other than contraception, as well as women who presented any medical contraindications to the use of COCs.

This study was approved by the institutional review board of the Gonçalo Moniz Institute, Oswaldo Cruz Foundation (IGM-FIOCRUZ—Bahia—Brazil) and was conducted in compliance with the ethical principles established by the revised Declaration of Helsinki. Prior to enrollment in the study, a term of informed written consent was obtained from all research participants, or from the parents/legal guardians of all non-adult participants.

### Data Collection

Data regarding the current generation of COC use and length of use, as well as demographic variables, reproductive, and health history, were collected using a standardized and confidential questionnaire (self-reported) at the time of enrollment, in addition to anthropometric parameters. Height was measured using a fixed scale (1 cm accuracy) with shoulders placed in a normal position. Weight was obtained by an electronic digital weighing scale (100 g accuracy) while women were dressed in light clothing without shoes. BMI was calculated by dividing weight (in kilograms) by the square of height (in meters). For the measurement of waist circumference, a non-flexible tape with 0.1 cm accuracy was positioned along the smallest area at the end of expiration, without any compulsory pressure. BP readings were taken using an Omron Model M7 digital automatic BP monitor (Omron Healthcare, Inc., Lake Forest, IL, USA) after the participant had been in a relaxed, seated position for at least 5 min. Three BP readings were obtained at 5-min intervals, and the average value of these measurements was used in our analyses.

### Biological Analyses

Blood samples were collected at the time of enrollment by venipuncture the morning after 12 h of fasting under standardized conditions. Biochemical analyses were performed in serum, and fibrinogen was analyzed in plasma (12).

Glucose, iron, total cholesterol, high-density lipoprotein cholesterol (HDL-c), and triglycerides were analyzed in fresh serum samples using automated equipment (A25 Biosystems S.A., Costa Brava, Barcelona). Low-density lipoprotein cholesterol (LDL-c) and very low-density lipoprotein levels were determined by the Friedewald equation, except for cases in which triglycerides values exceeded 400 mg/dL. C-reactive protein (CRP) levels were measured in fresh samples using a high-sensitivity assay (Beckman Coulter, Inc., Fullerton, CA, USA). Plasma fibrinogen was measured on a Destiny Plus analyzer (Tcoag, TriniCLOT, Trinity Biotech, Ireland) using the coagulometric method. Ferritin serum levels were acquired using automated equipment (Access 2 Beckman Coulter, Inc., Fullerton, CA, USA). Nitric oxide metabolites (NOm) were investigated by the Griess method, following a previously described protocol (13).

Biological assays were performed at the Laboratory of Hematology, Genetic and Computational Biology of IGM-FIOCRUZ and at the Clinical Analyses Laboratory of the College of Pharmaceutical Sciences, UFBA.

### Statistical Analysis

All statistical analyses were performed using the IBM Statistical Package for the Social Sciences (SPSS) version 21.0 (Armonk, NY, USA) and GraphPad Prism version 6.0 (GraphPad software, San Diego, CA, USA) considering *p* < 0.05 as significant. Normal distribution analyses of the quantitative variables were performed using the Kolmogorov–Smirnov and Shapiro–Wilk tests. The independent *t*-test and Mann–Whitney *U* test were used to compare the median value of two groups, in accordance with the distribution of each variable of interest. Odds ratios and confidence intervals (95% CI) were calculated considering CVD risk factors (5). Multivariate analysis was performed to estimate the likelihood of having an HDL-c alteration as the outcome (dependent variable) and any possible associations with second-generation progestin COCs, fibrinogen, CRP, or triglycerides (independent variables), which was adjusted for age and duration of COC use.

## Results

Among women who were COC users, 19.1% (93/481) were classified as OB and 79.9% (388/481) as NOB (normal or overweight), while among women in the COC non-users group, 19.7% (21/110) were OB and 80.3% (89/110) were NOB.

Data regarding the clinical and laboratory parameters of OB and NOB women in the COC users and non-user groups are described in Table 1. When we compared NOB women who were COC users (388/481) with non-users (89/481) (P1), COC users had increased levels of fasting glucose (NOB COC users: 87.0 mg/dL; NOB COC non-users: 79.0 mg/dL; *p* < 0.001), LDL-c (NOB COC users: 103.5 mg/dL; NOB COC non-users: 79.5 mg/dL; *p* < 0.001), VLDL-c (NOB COC users: 18.6 mg/dL; NOB COC non-users: 15.4 mg/dL; *p* = 0.002), triglycerides (NOB COC users: 93.0 mg/dL; NOB COC non-users: 76.0 mg/dL; *p* = 0.001), CRP (NOB COC users: 5.0 mg/L; NOB COC non-users: 1.8 mg/L; *p* < 0.001), and NOm (NOB COC users: 27.0 μM; NOB COC non-users: 18.7 μM; *p* < 0.001). Decreased levels of HDL-c (NOB COC users: 53.0 mg/dL; NOB COC non-users: 75.0 mg/dL; *p* < 0.001) were verified among COC users.

**Table 1:** Clinical and laboratory parameters of non-obese and obese women who were combined oral contraceptive users or not.

	NOB, COCs^−^ (*N* = 89)	NOB, COCs^+^ (*N* = 388)	OB, COCs^−^ (*N* = 21)	OB, COCs^+^ (*N* = 93)	P1	P2	P3	P4
				
	Median (25th–75th)	Median (25th–75th)	Median (25th–75th)	Median (25th–75th)	*p*-Values			
Age (years)	32.5 (21.0–40.0)	29.0 (23.0–35.0)	37.0 (32.0–39.3)	32.0 (27.0–37.5)	0.2	0.07	**0.003**	0.07
Duration of use (months)	–	24.0 (6.0–78.0)	–	24.0 (6.0–49.0)	–	–	–	–
SBP (mmHg)	110.0 (103.0–118.8)	110.0 (100.0–120.0)	115.0 (110.0–127.5)	120.0 (110.0–130.0)	0.9	0.8	**<0.001**	**<0.001**
DBP (mmHg)	70.0 (63.0–78.0)	70.0 (60.0–71.5)	76.5 (70.0–84.5)	80.0 (70.0–80.0)	0.5	0.8	**<0.001**	**0.001**
Blood glucose (mg/dL)	79.0 (73.3–84.0)	87.0 (82.3–95.0)	86.5 (83.5–91.8)	89.0 (83.5–98.5)	**<0.001**	0.1	**0.03**	**<0.001**
Total cholesterol (mg/dL)	171.5 (155.3–189.5)	178.0 (157.0–202.0)	200.0 (171.0–211.5)	184.0 (165.0–203.5)	0.1	0.3	0.1	**0.008**
HDL-cholesterol (mg/dL)	75.0 (63.0–87.0)	53.0 (45.0–61.0)	71.0 (65.8–76.5)	47.0 (42.0–55.0)	**<0.001**	**<0.001**	**<0.001**	**<0.001**
LDL-cholesterol (mg/dL)	79.5 (65.8–97.0)	103.5 (85.5–126.6)	102.8 (67.3–120.2)	105.4 (93.7–129.5)	**<0.001**	0.2	0.3	**<0.001**
VLDL-cholesterol (mg/dL)	15.4 (12.3–21.3)	18.6 (14.0–24.2)	20.0 (15.5–25.1)	25.8 (15.7–30.6)	**0.002**	0.3	**<0.001**	**<0.001**
Triglycerides (mg/dL)	76.0 (61.0–106.5)	93.0 (70.0–121.0)	103.0 (78.8–133.8)	129.0 (78.5–153.0)	**0.001**	0.5	**<0.001**	**<0.001**
Ferritin (ng/dL)	58.3 (22.1–97.0)	52.0 (29.0–83.7)	45.2 (22.9–86.1)	67.4 (38.3–151.2)	0.9	**0.02**	**<0.001**	**0.006**
Fibrinogen (mg/dL)	329.0 (256.0–407.0)	270.0 (197.0–337.0)	316.5 (223.3–389.8)	277.0 (205.3–380.3)	0.06	0.5	0.3	**0.029**
CRP (mg/L)	1.8 (1.1–3.3)	5.0 (3.0–8.0)	5.4 (2.6–12.0)	9.0 (5.9–15.0)	**<0.001**	**0.04**	**<0.001**	**<0.001**
NOm (μM)	18.7 (9.7–21.9)	27.0 (16.5–40.4)	21.1 (18.4–23.1)	31.7 (18.3–51.3)	**<0.001**	**0.01**	**0.02**	**<0.001**

*Significant *p* values (<0.05) are shown in bold; Mann–Whitney *U* test. P1 = NOB COCs^−^ X NOB COCs^+^; P2 = OB COCs^−^ X OB COCs^+^; P3 = NOB COCs^+^ X OB COCs^+^; P4 = NOB COCs^−^ X OB COCs^+^*.

*COCs^+^, combined oral contraceptive users; COCs^−^, combined oral contraceptive non-users; NOB, non-obese; OB, obese; SBP, systolic blood pressure; DBP, diastolic blood pressure; HDL-c, high-density lipoprotein-cholesterol; LDL-c, low-density lipoprotein-cholesterol; VLDL-c, very low-density lipoprotein-cholesterol; CRP, C-reactive protein; NOm, nitric oxide metabolites*.

Additionally, we compared OB women who were COC users (93/481) with non-users (21/110) (Table 1—P2). The following parameters were found to be significantly altered in OB COC users: ferritin (OB COC users: 67.4 ng/dL; OB COC non-users: 45.2 ng/dL; *p* = 0.02), CRP (OB COC users: 9.0 mg/L, OB COC non-users: 5.4 mg/L; *p* = 0.04), and NOm (OB COC users: 31.7 µM, OB COC non-users: 21.1 μM; *p* = 0.01). HDL-c levels were lower in OB COC users (OB COC users: 47.0 mg/dL; OB COC non-users: 71.0 mg/dL; *p* < 0.001).

Clinical and laboratory parameters were also compared among OB (93/481) and NOB (388/481) women who were COC users (Table 1—P3). Significantly higher values were observed in OB women compared to NOB: age (OB COC users: 32.0 years; NOB COC users: 29.0 years; *p* = 0.003), systolic blood pressure (SBP) (OB COC users: 120.0 mmHg; NOB COC users: 110.0 mmHg; *p* < 0.001), diastolic blood pressure (DBP) (OB COC users: 80.0 mmHg; NOB COC users: 70.0 mmHg; *p* < 0.001), fasting glucose (OB COC users: 89.0 mg/dL; NOB COC users: 87.0 mg/dL; *p* = 0.03), VLDL-c (OB COC users: 25.8 mg/dL; NOB COC users: 18.6 mg/dL; *p* < 0.001), triglycerides (OB COC users: 129.0 mg/dL; NOB COC users: 93.0 mg/dL; *p* < 0.001), ferritin (OB COC users: 67.4 ng/dL; NOB COC users: 52.0 ng/dL; *p* < 0.001), CRP (OB COC users: 9.0 mg/L; NOB COC users: 5.0 mg/L; *p* < 0.001), and NOm (OB COC users: 31.7 µM; NOB COC users: 27.0 µM; *p* = 0.02). HDL-c (OB COC users: 47.0 mg/dL; NOB COC users: 53.0 mg/dL; *p* < 0.001) levels were found to be significantly lower in OB COC users.

Finally, we compared OB women who were COC users (93/481) with NOB COC non-users (89/110) (Table 1—P4). Almost all the parameters evaluated were significantly higher (*p* < 0.05) among OB women who were COC users than NOB COC non-users, while HDL-c (*p* < 0.001) and fibrinogen (*p* = 0.029) were significantly lower in the OB COC users. Despite the alterations observed, only CRP levels (OB COC users: 9.0 mg/L, NOB COC non-users: 1.8 mg/L; *p* < 0.001) and HDL-c levels (OB COC users: 47.0 mg/dL; NOB COC non-users: 75.0 mg/dL; *p* < 0.001) were determined to be outside normal reference ranges: CRP (>3 mg/dL), HDL-c (<50 mg/dL). These are considered markers of CVD risk.

The influence of duration of COC use, age, and biochemical parameters was also verified among all women who were COC users (data not shown). No significant associations were found between these parameters and the duration of COC use (*p* > 0.05).

**Figure 1:**
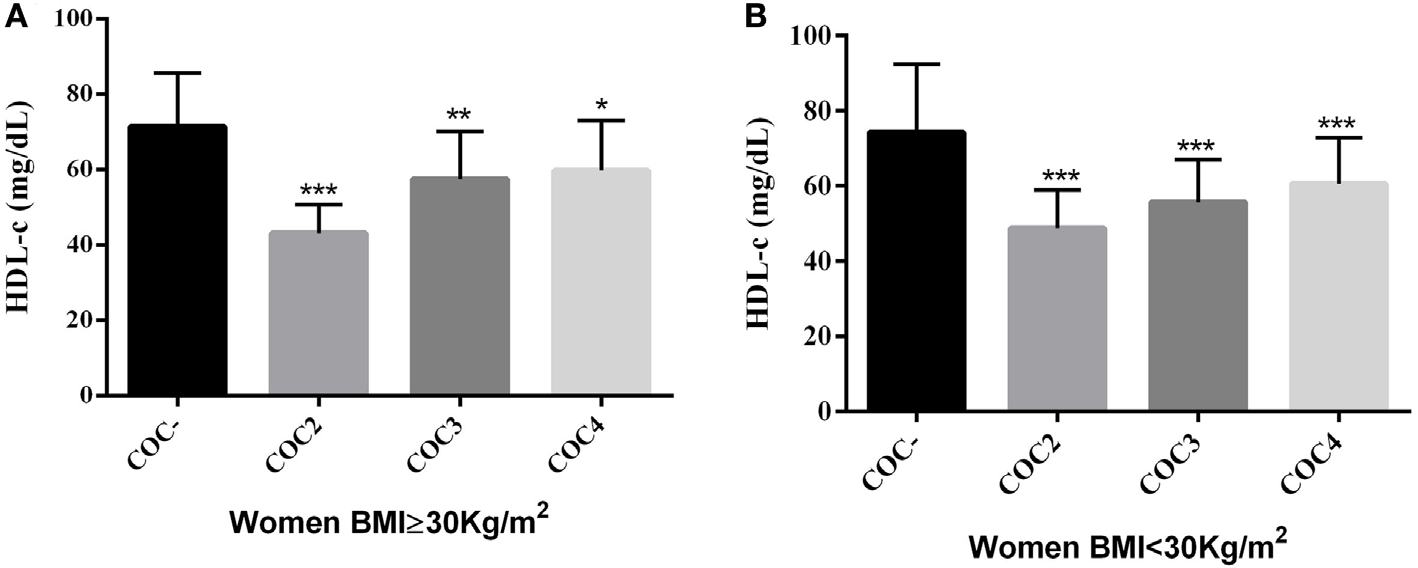
**(A)** Comparison of HDL-c levels in obese women (BMI ≥ 30 kg/m^2^) using COCs with second, third, and fourth generations of progestins with that in women who were COC non-users *p < 0.001, **p = 0.002, ***p = 0.04. Mann–Whitney *U* test; **(B)** Comparison of HDL-c levels in women with BMI < 30 kg/m^2^ using COCs with second, third, and fourth generations of progestins with that in women who were COC non-users, ***p < 0.001. Mann–Whitney *U* test. HDL-c, high-density lipoprotein-cholesterol; BMI, body mass index; COC^−^, women COCs non-users; COC2, second generations combined oral contraceptives; COC3, third generations combined oral contraceptives; COC4, fourth generations combined oral contraceptives.

**Figure 2:**
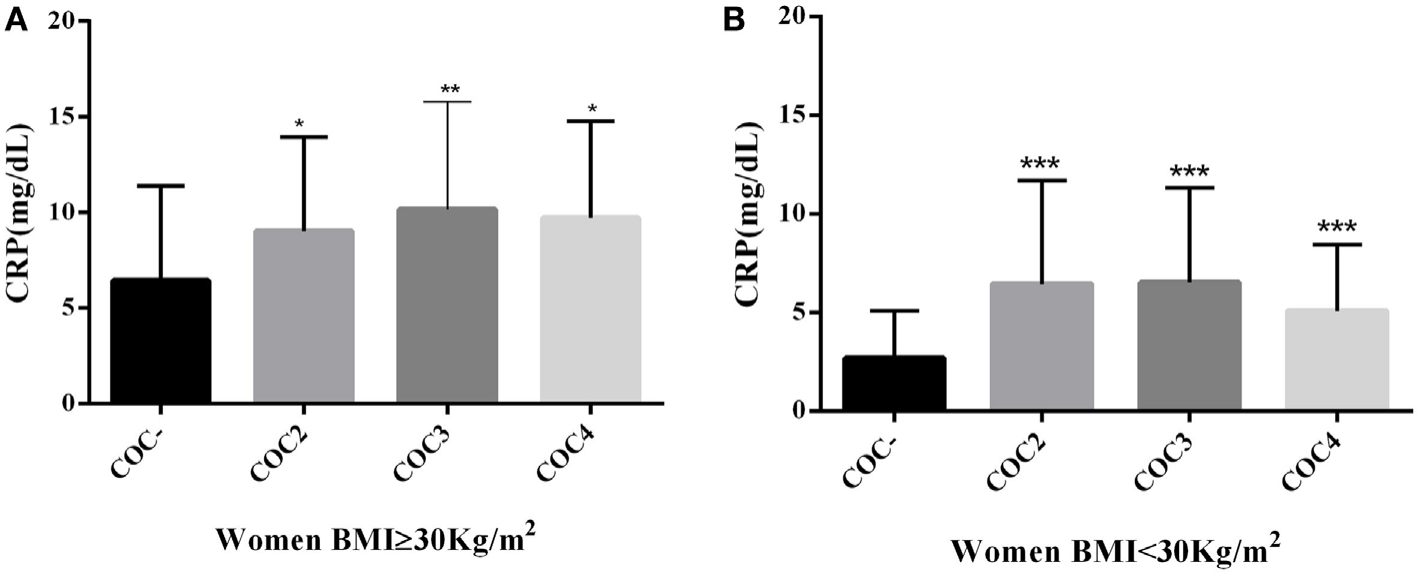
**(A)** Comparison of CRP levels in obese women (BMI ≥ 30 kg/m^2^) using COCs with second, third, and fourth generations of progestins with that in women who were COC non-users, *p = 0.02, **p = 0.04. Mann–Whitney *U* test; **(B)** Comparison of CRP levels in women with BMI < 30 kg/m^2^ using COCs with second, third, and fourth generations of progestins with that in women who were COC non-users, ***p < 0.001. Mann–Whitney *U* test. CRP, C-reactive protein; BMI, body mass index; COC^−^, women COCs non-users; COC2, second generations combined oral contraceptives; COC3, third generations combined oral contraceptives; COC4, fourth generations combined oral contraceptives.

We evaluated these same laboratory parameters among OB women who used different generations of COCs [second generation: levonorgestrel plus ethinylestradiol (EE); third generation: desogestrel or gestodene plus EE; fourth generation: drospirenone or cyproterone plus EE]. We observed that all generations of COCs were associated with decreased HDL-c levels (*p* < 0.05) among OB women who were COC users compared to non-users (Figure 1). However, only second-generation progestin pills were associated with HDL-c levels under normal reference range limits (HDL-c ≤ 50 mg/dL). CRP also presented significant differences between the studied groups, with the highest levels seen in COCs users (*p* < 0.05), regardless of COC generation (Figure 2).

We also evaluated COC use, obesity or both in relation to cardiovascular risk factors categorized according to the American Heart Association (5), considering HDL-c ≤ 50 mg/dL, CRP ≥ 3 mg/L, fibrinogen ≥ 350 mg/dL, SBP ≥ 140 mmHg, DBP ≥ 90 mmHg, and total cholesterol ≥ 240 mg/dL (Table 2). We observed that CVD risk was higher among OB women who were COC users than among NOB COC non-users with respect to HDL-c ≤ 50 mg/dL (*p* < 0.001), CRP ≥ 3 mg/dL (*p* < 0.001), and SBP ≥ 140 mmHg (*p* < 0.001) (Table 2). In addition, NOB women who were COC users had 5.8-fold odds of having HDL-c ≤ 50 mg/dL, while OB women who were COC users had 14.5-fold odds as compared to NOB COC non-users. Furthermore, the odds of having CRP ≥ 3 mg/dL was 8.2- and 39.8-fold higher in NOB and OB women who were COC users, respectively, in comparison to NOB COC non-users.

**Table 2:** Parameters of cardiovascular risk in women of reproductive age considering the oral contraceptive use and obesity.

	NOB, COCs^−^ (*N* = 89)		NOB, COCs^+^ (*N* = 388)		OB, COCs^−^ (*N* = 21)		OB, COCs^+^ (*N* = 93)	
	*N* (%)	OR (95% CI)	*N* (%)	OR (95% CI)^a^	*N* (%)	OR (95% CI)^b^	*N* (%)	OR (95% CI)^c^
HDL^CVR^	9 (10.2)	–	153 (39.4)	5.8 (2.8–11.9)	3 (14.3)	1.5 (0.4–6.0)*	58 (62.4)	14.5 (6.5–32.6)
CRP^CVR^	27 (30.3)	–	303 (78.1)	8.2 (4.9–13.7)	15 (71.4)	5.7 (2.0–16.4)	88 (94.6)	39.8 (14.5–109.0)
Fibrinogen^CVR^	20 (22.5)	–	51 (13.1)	0.5 (0.3–0.9)	8 (38.1)	2.1 (0.8–5.8)	29 (31.2)	1.6 (0.8–3.2)
SBP^CVR^	2 (2.2)	–	29 (7.5)	3.5 (0.8–15.0)*	1 (4.8)	2.2 (0.2–25.2)*	16 (17.2)	9.6 (2.2–43.0)*
DBP^CVR^	0 (0)	–	31 (8.0)	–	3 (14.3)	–	14 (15.1)	–
Total cholesterol^CVR^	3 (3.4)	–	19 (4.9)	1.5 (0.4–5.1)*	2 (9.5)	3.0 (0.5–19.3)*	4 (4.3)	1.3 (0.3–5.9)*

*^a^NOB COCs^−^ X NOB COCs^+^*.

*^b^NOB COCs^−^ X OB COCs^−^*.

*^c^NOB COCs^−^ X OB COCs^+^*.

*significate the statistic test χ^2^

*COCs^+^, combined oral contraceptive users; COCs^−^, combined oral contraceptive non-users; NOB, non-obese; OB, obese; HDL, high-density lipoprotein; CRP, C-reactive protein; SBP, systolic blood pressure; DBP, diastolic blood pressure; OR, odds ratio; CI, confidence interval. HDL^CVR^, defined as HDL ≤ 50 mg/dL; CRP^CVR^, defined as CRP ≥ 3 mg/L; Fibrinogen^CVR^, defined as fibrinogen ≥ 350 mg/dL; SBP^CVR^, defined as SBP ≥ 140 mmHg; DBP^CVR^, defined as DBP ≥ 90 mmHg; Total cholesterol^CVR^, defined as total cholesterol ≥ 240 mg/dL*.

Our multivariate analysis, adjusted for age and duration of COC use, showed that second-generation progestin COC use was independently associated with levels of HDL-c ≤ 50 mg/dL (*p* < 0.001; OR = 8.9; 95% CI 2.8–28.9) (Table 3). No significant associations were detected with respect to the other independent variables considered.

**Table 3:** Influence of COCs progestin generation in HDL-c levels.

Variables	*B*	SE	Wald test	*p*-Value	OR	95% CI
Fibrinogen^a^	0.071	0.587	0.015	0.9	1.1	0.3–3.4
Triglycerides^b^	0.791	0.689	1.317	0.3	2.2	0.6–8.5
CRP^c^	0.138	1.238	0.012	0.9	1.1	0.1–13.0
COCs second generation	2.195	0.597	13.520	**<0.001**	8.9	2.8–28.9

*p < 0.05 in bold*.

*HDL-c as a dependent variable defined as HDL-c ≤ 50 mg/dL*.

*^a^Defined as fibrinogen ≥ 350 mg/dL*.

*^b^Defined as triglycerides ≥ 150 mg/dL*.

*^c^Defined as CRP ≥ 3 mg/L*.

*HDL-c, high-density lipoprotein-cholesterol; COCs, combined oral contraceptive; LDL-c, low-density lipoprotein-cholesterol; CRP, C-reactive protein; SE, standard error; B, coefficient; OR, odds ratio; CI, confidence interval*.

## Discussion

This study was conducted due to the increasing prevalence of obesity among adolescents and adult women who potentially may use COCs. Both obesity and the use of oral contraceptives can impact biochemical and inflammatory profiles, mainly markers associated with cardiovascular risk, which may lead to dangerous repercussions in the quality of life of these women. Obesity was observed among 19.1% of women COC users and 19.7% among non-users. Farahmand et al. (14) reported that 23% of obese Iranian women were COC users (14). Pomp et al. (15) verified that at the time of first venous thrombosis, 21% of Dutch women were obese, and in these authors’ control group, 13% were obese (MEGA study) (15). In a cohort study involving American women COC users, Hurwitz et al. (16) reported obesity rates ranging from 17.7 to 32.4% (16). Obesity has been reported to predispose individuals to CVD and VTE after an increase in procoagulant factors, such as fibrinogen, circulating microparticles, factor VII, factor VIII, factor IX, and factor XII (17, 18, 19). Furthermore, obesity confers a proinflammatory status and leads to increased CRP levels, an acute phase protein predictor of CVD risk. In addition, obesity can provoke adverse effects on arterial walls and systemic inflammation (20). Additionally, COCs use also increases the risk of VTE and CVD both independently and synergistically in association with obesity (15, 21–24).

Our study comparing women who were COC users as well as non-users, whether obese or not, showed significant differences among the studied groups, especially with respect to lipid and inflammatory parameters. CRP, an inflammatory acute phase protein and a marker of low-grade chronic inflammation (24, 25), was increased among obese women regardless of COCs use. CRP levels were also found to be surprisingly higher among COC users than non-users, which suggests a synergic effect between obesity and COCs use. The present study found significantly elevated CRP levels, especially among obese women who were COCs users, that were higher than 3 mg/dL, a borderline value that is associated with the development of CVD (26). Additionally, increased CRP levels were significantly associated with all types of progestin, especially third-generation progestins that provided beneficial effects on lipid parameters, thusly reducing cardiovascular risk (27). Kluft et al. (28) also reported a continuous and rather strong increase in CRP levels among healthy women who used COCs during 12 cycles. These authors suggested that the pro-inflammatory impact of COCs use may explain the increased risk of thromboembolic adverse effects in these women (28). Williams et al. (24), in a cross-sectional study including obese women who used COCs, reported elevated CRP levels related to COCs, regardless of progestin type (24). Dreon et al. (29) also noted a significant association of low-dose COCs with higher levels of CRP (29). Increased production of some cytokines, such as interleukin (IL)-6 and tumor necrosis factor alpha (TNF-α), mediators of inflammation that affect endothelial function, has been associated with increases in CRP concentrations that may contribute to the atherogenic process. In addition, it is possible that increased cytokine secretion in adipose tissue in obese individuals results in elevated CRP secretion by the liver (30, 31).

Ferritin, an indicator of iron deposits, is also an inflammatory acute phase protein that was analyzed in our study. The isolated use of COCs does not seem to interfere with ferritin levels. Nevertheless, in our study, obesity together with COC use was associated with high ferritin levels, suggesting a synergic effect. Alam et al. (32) found that obese individuals exhibited high levels of serum ferritin and CRP, attributing these results to the inflammatory status produced by increased adipose tissue (32). While the inflammatory condition induced by obesity and COC use may synergistically affect the hepatic synthesis of ferritin, the underlying mechanism pertaining to the influence of COCs on ferritin levels has not yet been elucidated.

Increased NOm levels was also found to be associated with obesity, isolated COC use, or a combination of both, since elevated levels was observed in all of the groups that we analyzed. Although estrogens lead to increases in nitric oxide (NO) levels due to the stimulation of endothelial NO synthase, which controls the normal functioning of the endothelium, certain progestins counteract this effect by developing an endothelial alteration that may increase CVD risk (33, 34, 35). Zerr-Fouineau et al. (35) reported that progestins, such as levonorgestrel, had no influence in NO production by endothelial cells stimulated with 17β-estradiol (35). Herein, we measured overall NOm, which may have been released by estrogen stimulation, but NOm is also known to be produced in high concentrations during pathologic conditions, in addition to inflammatory processes (36, 37). Our results indicate that obese women who were COC users may present an inflammatory condition due to the elevated levels of CRP and ferritin found in our study. Accordingly, increases in Nom may also be related to nitrosative stress.

Regarding our lipid profile analysis, we verified that the combination of COCs use and obesity presents a synergistic effect in the reduction of HDL-c to below-average levels among women who used COCs. Normal HDL-c levels are implicated in the inhibition of atherosclerosis, since antioxidant and anti-inflammatory properties have been demonstrated in *in vitro* studies (38). Individuals with increased HDL-c levels have also been reported to have a reduced incidence of CVD (39). Furthermore, HDL-c concentrations are known to directly attenuate tissue factor, selectins expression and platelet activation, in addition to indirectly promoting the inhibition of thrombosis (39, 40).

The interference of COCs use on an individual’s lipids profile is related to formulation type, i.e., androgenic, antiandrogenic, estrogenic, or antiestrogenic. The progestin component of COCs, e.g., desogestrel and gestodene, has been reported to raise HDL-c and decrease LDL-c due to their antiandrogenic effect (4). We found low levels of HDL-c among obese women who were second-generation COC users compared with higher levels in women who used later generations, which implied an increased association of CVD risk. Greenlund et al. (41) studied the overall influence of COCs on serum lipids among non-obese women initiating contraceptive use and reported a significant decrease in HDL-c levels among COC users compared with non-users in the period from 1985 to 1986, in contrast to no significant difference seen among those women analyzed from 1988 to 1991 (41). Wiegratz et al. (42) analyzed the impact on HDL-c and HDL2-c levels after six cycles of different types of COCs use, reporting significant decreases in women treated with a combination containing EE and levonorgestrel (42).

Agren et al. (43) compared the effects of COCs employing nomegestrol, 17β-estradiol, or EE plus levonorgestrel after six treatment cycles and verified, in comparison to baseline levels, significant decreases in HDL-c in addition to increases in LDL-c and triglycerides among women using the combined formulation with levonorgestrel (43).

According to a study performed by Greenlund et al. (41), the effect of COCs on lipid profiles seems to be immediate, yet reversible (41). Because our results demonstrated a synergistic influence of COCs and obesity on decreases in HDL-c levels, the reversibility of these effects may be called into question due to the inflammatory conditions observed in obese women. However, further studies are necessary to explain this observation.

Additionally, it has been previously reported that HDL-c levels are inversely related to circulating levels of CRP (44, 45). Our results showed that obese women who were COC users had above-average CRP levels and decreased HDL-c levels, which constitutes a possible inflammatory scenario that corroborates previous data.

Our data reinforce the unfavorable association between COCs use and obesity, as evidenced by the impact on cardiometabolic parameters, especially with respect to second-generation COCs formulations (4, 42, 43, 46). While we must take into consideration the limitations related to classifying obesity using BMI measurements, this method is inexpensive, reliable and performed with facility. Another limitation of our study is that we were not able to distinguish whether the detected alterations in lipid and inflammatory parameters are directly the result of obesity or COC use, due to the cross-sectional nature of the present study.

In conclusion, the present study demonstrated a clear association between obesity and COC use on alterations in metabolic parameters, notably HDL-c and CRP, which are known to contribute to the development of CVD. Moreover, COC use, especially formulations employing second-generation progestins, was independently associated with low levels of HDL-c. In light of the need to prevent unintended pregnancy among obese women, it is imperative for health professionals to select the most effective and safe contraception methods for these women, taking into consideration WHO recommendations, including progestogen-only pills, injectable contraceptives (containing depot medroxyprogesterone acetate or norethisterone enanthate), and intrauterine devices (6). In addition, obese women should receive counseling regarding weight loss and preventive health care to decrease CVD and VTE risks. Moreover, it is also important to consider the presence of other CVD conditions, such as hypertension and diabetes, in obese women when prescribing contraceptive methods.

## Ethics Statement

This study was approved by the ethics committee of the “Gonçalo Moniz Institute, Oswaldo Cruz Foundation (IGM-FIOCRUZ)” and was conducted in compliance with the ethical principles of the revised Declaration of Helsinki. Before enrolment into the study, informed written informed consent was obtained from all of the women. Data were collected from August 2013 to July 2014.

## Author Contributions

JF, MA, and EA contributed to the study concept and design, collected data, supervised the study, participated in data analysis and interpretation, and drafted, reviewed, and edited the manuscript. CF, LV, RS, CG, and CB contributed to the study concept and design, collected data, participated in data analysis and interpretation, and reviewed and edited the manuscript. RO contributed to the study concept and design, participated in data analysis and interpretation, and reviewed and edited the manuscript. MG contributed to the study concept and design, supervised the study, participated in data analysis and interpretation as well as reviewed, and edited the manuscript.

## Conflict of Interest Statement

The authors declare that the research was conducted in the absence of any commercial or financial relationships that could be construed as a potential conflict of interest. The reviewer SP and handling editor declared their shared affiliation, and the handling editor states that the process nevertheless met the standards of a fair and objective review.

## Abbreviations

BMI, body mass index; COC, combined oral contraceptives; CRP, C-reactive protein; HDL-c, high-density lipoprotein cholesterol; LDL, low-density lipoprotein cholesterol; VLDL, very low-density lipoprotein cholesterol; EE, ethinylestradiol; LEVO, levonorgestrel; CVD, cardiovascular disease, OB, obese; NOB, non-obese. SBP, systolic blood pressure; DBP, diastolic blood pressure; NO, nitric oxide; NOm, nitric oxide metabolites.
